# Advances in research on spexin-mediated regulation of reproductive function in vertebrates

**DOI:** 10.3389/fendo.2024.1422711

**Published:** 2024-06-10

**Authors:** Xiaojing Chen, Yuyan Feng, Shudi Dai, Binbin Guo, Leyan Yan, Jie Liu, Huanxi Zhu

**Affiliations:** ^1^Key Laboratory of Animal Physiology and Biochemistry, Ministry of Agriculture and Rural Affairs, College of Veterinary Medicine, Nanjing Agricultural University, Nanjing, China; ^2^School of Life Science, Jiangsu University, Zhenjiang, China; ^3^Key Laboratory of Crop and Livestock Integration, Ministry of Agriculture, Institute of Animal Science, Jiangsu Academy of Agricultural Sciences, Jiangsu Province Engineering Research Center of Precision Animal Breeding, Nanjing, China

**Keywords:** spexin, HPG axis, hypothalamus, pituitary, ovary

## Abstract

Spexin (SPX, NPQ) is a 14-amino acid neuroactive peptide identified using bioinformatics. This amino acid sequence of the mature spexin peptide has been highly conserved during species evolution and is widely distributed in the central nervous system and peripheral tissues and organs. Therefore, spexin may play a role in various biological functions. Spexin, the cognate ligand for GALR2/3, acting as a neuromodulator or endocrine signaling factor, can inhibit reproductive performance. However, controversies and gaps in knowledge persist regarding spexin-mediated regulation of animal reproductive functions. This review focuses on the hypothalamic-pituitary-gonadal axis and provides a comprehensive overview of the impact of spexin on reproduction. Through this review, we aim to enhance understanding and obtain in-depth insights into the regulation of reproduction by spexin peptides, thereby providing a scientific basis for future investigations into the molecular mechanisms underlying the influence of spexin on reproductive function. Such investigations hold potential benefits for optimizing farming practices in livestock, poultry, and fish industries.

## 1 Introduction

**Figure 1:**
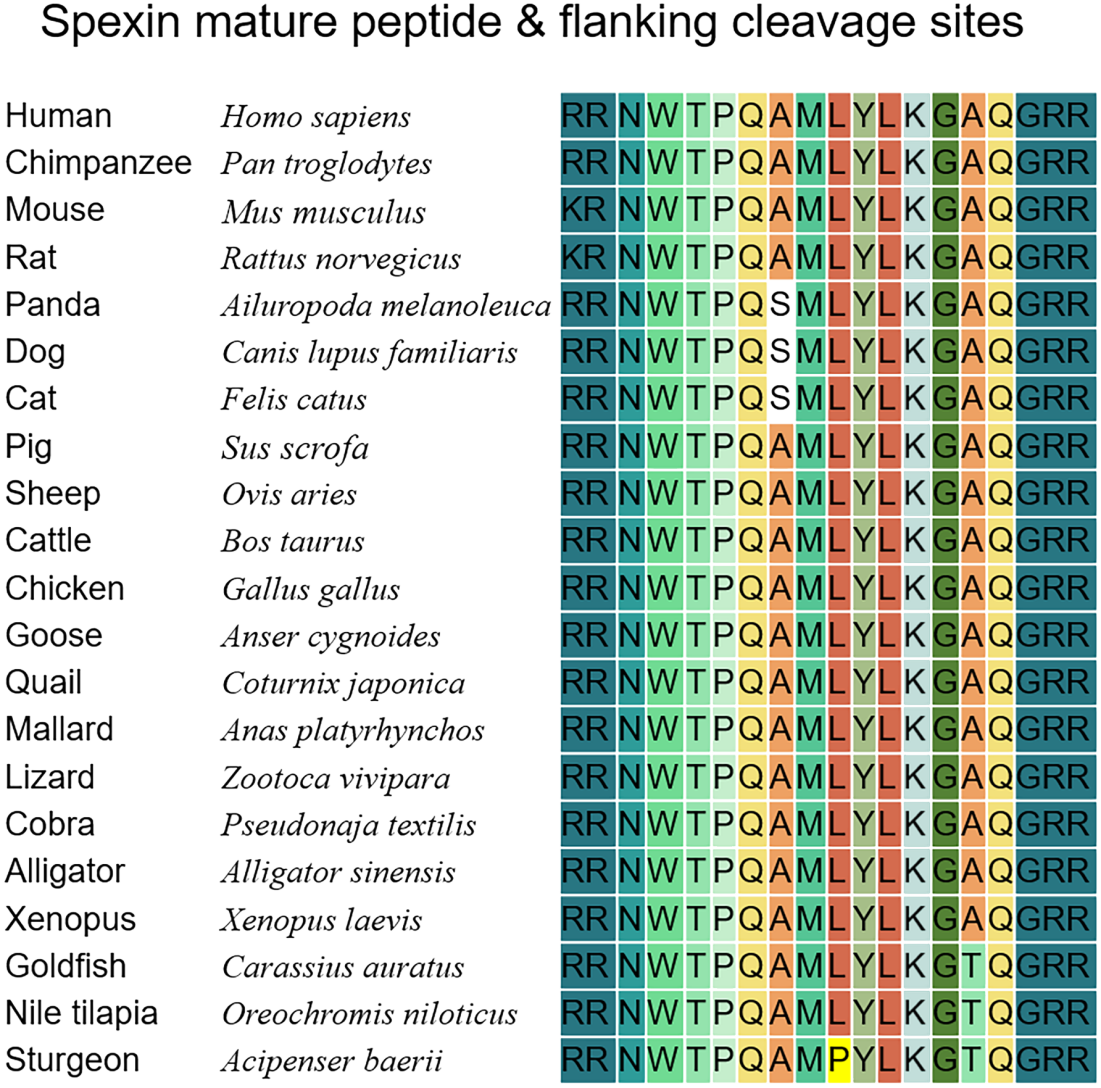
Protein sequences of mature spexin in vertebrate species. The dibasic cleavage sites on both sides of the mature peptide of spexin are marked with dark blue. Every amino acid of the spexin is colored in different color.

Spexin (SPX, NPQ) is a novel endogenous neuropeptide that was first discovered in 2007 via bioinformatic analysis based on hidden Markov models (1). In the human genome, this neuropeptide is encoded by *C12ORF39* located on chromosome 12 and is also referred to as C12ORF39 (2). Spexin is not only found in humans but also in other mammals (such as rodents (3, 4), cattle (5), sheep (6), and pigs (7)) and non-mammalian vertebrates (such as teleosts and birds) (8). The spexin precursor proteins of humans, mice, and rats include a signal peptide sequence, two cleavage sites, and a highly conserved mature peptide sequence. The conserved sequence between two cleavage sites is NWTPQAMLYLKGAQ, known as spexin (4). The mature spexin peptide is highly conserved across species (*https://www.ncbi.nlm.nih.gov/* ), with some variations at position 13 (Ala^13^ replaced with Thr^13^) in teleost species (9–11); position 6 (Ala^6^ replaced with Ser^6^) in dogs, cats and pandas (12); and positions 8 and 13 (Leu^8^ replaced with Pro^8^ and Ala^13^ replaced with Thr^13^) in sturgeon (**Figure 1**). Homologous paralog forms of *spx*, namely *spx1a*, *spx1b*, and *spx2*, have been found in some birds and fish. *Spx2* has been reported in *Xenopus tropicalis*, chickens, zebrafish (3), and half-smooth tongue sole (13). Nile tilapia and other cichlid fish species have two *spx1* paralogs (*spx1a* and *spx1b*), but no *spx2* (14). *Spx2* has not been previously detected in mammals (3). Not all spexin-2 peptides exhibit tetradeceptide properties, and in *Cynoglossus semilaevis* (in half-smooth tongue sole), spexin-2 comprises 17 amino acids (13). Spexin and its receptors, GALR2 (GALR2a and 2b)/3, are widely expressed in a variety of systems and tissues, including the central nervous system, endocrine system, digestive system, reproductive system, muscles, epithelium, and fat. However, the widespread peripheral distribution varies across species at the mRNA transcript level (15). For example, ovarian expression in half-smooth tongue sole was lower than that in other fish. However, spexin is present in the brains of almost all species. Consistent with its wide tissue distribution, spexin is involved in multiple physiological activities, such as feeding and obesity, gastrointestinal peristalsis, glucose and lipid metabolism, pain regulation, and modulation of emotions and stress. Notably, *spx* and *kiss1* are located on the same chromosome and exhibit certain similarities in their mature peptide sequences, therefore, they are classified as members of the same peptide family (3, 16). Researchers initially speculated and confirmed the regulatory role of spexin in the reproductive axis of goldfish in 2013 (17). Considering the highly conserved sequence of spexin, we speculate that spexin may play an important role in reproduction. In recent years, research on reproductive regulation has been extended to other vertebrates. Although accumulating evidence highlights the significant regulatory function of spexin in puberty onset, gonadotropin secretion, gonadal development, and gamete maturation, numerous controversies and gaps persist regarding its regulatory role and signal transduction pathways across different species. The aim of this review was to comprehensively examine the role of spexin peptides in regulating reproduction, particularly focusing on their impact on the hypothalamic-pituitary-gonadal (HPG) axis. By compiling existing knowledge, we aim to enhance our understanding of the molecular mechanisms through which spexin influences reproductive function, laying the groundwork for future investigations.

## 2 Dynamic changes in spexin across reproductive stages

The expression of the *spx* gene shows dynamic changes during the breeding season and in different reproductive stages. To date, studies in this regard have primarily focused on teleosts. In teleosts, the expression of *spx* in the brain is highly dynamic throughout the reproductive stages, generally showing a progressive decrease with the progression of gonadal development cycles. Within one follicular maturation cycle in zebrafish, the expression of *spx* in the brain gradually increases during early follicular development (including primary growth and previtellogenesis) and then decreases during the late follicular stages (including early vitellogenesis, moderate vitellogenesis, full-grown, and germinal vesicle) (17). From October (non-breeding season) to February (breeding season), the expression of *spx* in female goldfish in southern China decreased. In addition, a sharp increase in the gonadosomatic index of female goldfish ovaries was noted (17). Gene expression of *spx* has been detected in the hypothalamus of spotted scat at ovarian developmental stages II, III, and IV, with the highest expression in stage II, moderate expression in stage III, and the lowest expression in stage IV (18). In addition, in the expression profile of hypothalamic *spx* of orange-spotted grouper, *spx* mRNA levels were high in the early developmental stages, from stage A (gonadal phase with gonadal primordium) to stage B (gonadal phase with incomplete ovarian lumen). Thereafter, the *spx* expression declined gradually during ovarian development, with low levels recorded at stage F when fully grown oocytes were observed in the breeding season (11). This progressive decline in *spx* expression throughout reproductive development suggests that spexin inhibits ovarian development and follicular maturation. Hence, it seems reasonable to hypothesize that high expression of *spx* may inhibit sexual maturity.

Furthermore, investigations into the dynamic expression patterns of *spx* have expanded to encompass other vertebrates. Liu et al. found that prolonged light exposure can promote egg laying in Yangzhou geese (19), and the latest research showed that prolonged exposure significantly reduces the expression of *spx* in the hypothalamus and pituitary gland of female Yangzhou geese, as well as the expression of *GALR2/3* in the pituitary gland of female (20). These results suggest a photoperiodic link between spexin and the HPG axis. However, there is no evidence of dynamic regulatory changes in spexin during seasonal reproduction in sheep (6). Notably, Zheng et al. (21) used transcription activator-like effector nucleases to design and establish a mutant zebrafish model and found that the reproductive phenotype of *spx1^−/−^* mutant zebrafish was not different from that of wild-type fish. The *spx1^−/−^* mutant fish still entered puberty normally, and the maturation of gametes was not affected. These data indicates that spexin is not essential for reproduction, and also partly explains species-specific differences in the role of spexin in puberty initiation and reproductive regulation.

## 3 Regulatory role of spexin on hypothalamic gonadotropin-releasing hormone

The HPG axis plays a crucial role in the regulation of reproductive functions in animals. GnRH/GnIH are the primary driving factors activating the gonadotropic axis in all vertebrates, regulating the puberty onset, follicular development, and gamete maturation by stimulating gonadotrophs in the anterior pituitary to synthesize and release luteinizing hormone (LH) and follicle-stimulating hormone (FSH) (22, 23). Spexin may act as a negative regulator of GnRH, leading to decreased GnRH release in the hypothalamus and potentially affecting reproductive performance. In a Yangzhou goose model of out-of-season breeding with a long photoperiod, prolonged light exposure reduced the expression of *spx* in the hypothalamus of geese while simultaneously increasing *gnrh* expression, confirming the association between GnRH and spexin (20). Wang et al. (24) confirmed the expression of *GALR2/3* in the mHypoA-GnRH/GFP cell line and speculated that palmitate increased the sensitivity of GnRH neurons to spexin by increasing the expression of *GALR2/3* at the mRNA level in these neurons, which was beneficial for the inhibition of reproduction by spexin. Furthermore, dual immunohistochemical experiments have demonstrated interactions between spexin and GnRH/GnIH neurons in certain regions of the brain and pituitary gland of sexually mature sea bass (25). Specifically, spexin-immunoreactive (ir) fibers (spexin-ir fibers) projected onto GnRH2-ir and GnIH-ir cells, whereas GnIH-ir and GnRH2-ir fibers projected onto spexin-ir cells. However, no co-localization of spexin-1 with GnRH1/3 has been observed (25). Moreover, spexin-1/2 may have distinct reproductive regulatory functions. Intraperitoneal injection of spexin-1 in half-smooth tongue sole increased the expression levels of hypothalamic *gnrh3* and *gnih*, whereas *gnrh2* transcript levels remained unaffected (13). Administration of spexin-2 appears to have no effect on *gnrh2/3* expression, suggesting that the GnRH system may not be an essential target of spexin-2 in reproduction (26). Interestingly, researchers have also investigated the effects of spexin-2 on the autocrine and paracrine regulation of the spexin system. Peripheral injection of spexin-2 downregulates the mRNA expression of *spx-1* with no effect on *spx-2* expression (26). However, despite the progressive downregulation of *spx* expression during ovarian structural and follicular development in orange-spotted grouper, peripheral injection of spexin does not affect the expression of *gnrh* (11). Collectively, the effects of spexin on GnRH neurons in several fish species remain controversial. Spexin has a possible effect on GnRH release when it directly acts on GnRH neurons via GALR2/3, probably acting as a balancing factor for GnRH neuronal activation, either remaining inactive or exerting an inhibitory effect on mRNA expression levels.

## 4 Regulation of pituitary gonadotropins by spexin

GnIH is an inhibitory factor of the reproductive axis, capable of suppressing the release of gonadotropins (27, 28). Spexin also tends to exert inhibitory effects in the reproductive field, and there are also studies shown that spexin can directly act on the pituitary gland to regulate the synthesis and release of gonadotropins. Liu et al. (17) found that serum LH levels decreased in goldfish intraperitoneally injected with spexin-14a, and *in vitro* experiments conducted on primary pituitary cells using heterologous radioimmunoassay (RIA) also showed a significant reduction in LH release from the pituitary gland after perfusion with spexin-14a. Notably, both *in vivo* and *in vitro* experiments involved the administration of a non-amidated C-terminal spexin-14. The C-terminal-free spexin-14 slightly inhibited the pituitary cell release of LH compared to spexin-14a, indicating that this C-terminal amidation is not essential for its biological activity (17). Similarly, Cohen et al. (14) found that intravenous injection of two spexin-1 paralogs (spexin-1a and spexin-1b) resulted in decreased levels of LH and FSH in the plasma of adult female tilapia. Additionally, when spexin-1a and spexin-1b were applied to LH cells in the pituitary slices, a reversible decrease in the action potential frequency of LH pituitary cells was observed, leading to a reduction in the amount of LH released. Therefore, a progressive decrease in *spx* expression during the reproductive period may contribute to the reproductive regulatory effects mediated by LH in teleost fish. In contrast, no inhibitory effect of spexin on LH has been observed in sheep, a classic mammalian model (6).

At the transcriptional level, existing data suggest that spexin may have significant species differences in its impact on reproductive activities mediated by the reproductive axis. In a long photoperiod-stimulated out-of-season breeding model, the expression of *spx* was downregulated in Yangzhou geese, whereas the expression of *lhβ* and *fshβ* was upregulated in the pituitary gland (20). Although the expression of *spx* in the brain of orange-spotted grouper decreased during the breeding season, administration of spexin both *in vivo* and *in vitro* did not significantly affect the expression of *lhβ* and *fshβ* in pituitary cells of orange-spotted grouper (11). In half-smooth tongue sole, intraperitoneal injection of spexin-1 and spexin-2 resulted in a decreased abundance of *gthα* mRNA in the pituitary gland. However, spexin-1 downregulated *fshβ*, whereas spexin-2 downregulated *lhβ* (13, 26). In summary, regardless of the paralogous type, spexin generally exhibits direct or indirect inhibitory effect on the pituitary gland and the release of gonadotropins in fish and birds, except for sheep.

## 5 Interactions between spexin and estradiol

GnRH neurons do not express estrogen receptor-alpha (ER-α). Hence, the feedback effect of estradiol (E2) on the reproductive axis may require upstream neurons (29). Kisspeptin neurons co-express ERs that are responsible for E2-mediated feedback (30, 31). The question arises whether spexin can potentially serve as pivotal mediators, such as kisspeptin. Currently, no studies have examined the molecular mechanisms by which estrogen acts on spexin neurons. Existing literature has only identified the regulation of spexin by E2. Liu et al. (17) showed a significant increase in *spx* expression in the hypothalamus of goldfish after ovariectomy (OVX group). Supplementation with an E2 injection in the OVX group (OVX+E2 group) decreased the expression of *spx* in goldfish, although it did not fully recover to preoperative levels. Deng et al. (18) found that E2 downregulated *spx* expression in the hypothalamus in a dose-dependent manner in an *in vitro* incubation test. The results of E2 injection under *in vivo* conditions were consistent with this finding. The downward trend in *spx* transcription levels was opposite to that of serum E2 levels during oocyte development from phase III to late phase IV in spotted scat. Therefore, Deng et al. (18) speculated that the dynamic decline in *spx* expression in the hypothalamus during ovarian development is regulated by E2 feedback. In contrast, the expression of *spx* in the hypothalamus of ewes was not affected by E2 (6). Spexin inhibited granulosa cells (GCs) proliferation and E2 release. Kurowska et al. (32)observed immunolocalization of spexin and GALR2/3 in human ovaries, including GCs, oocytes, theca, and cumulus cells. This finding aligns with previous data describing the expression of *spx* in rat ovaries (4). Studies on the molecular mechanism of spexin in the KGN cell line showed that the peptide negatively influenced GCs function by inhibiting cell proliferation via GALR2/3, MAP3/1, STAT3, and AKT and E2 secretion via GALR2/3, MAP3/1, and PKA (32). In summary, there is currently no direct evidence to suggest that spexin mediates E2 regulation of the reproductive axis, but limited research suggests that E2 regulates *spx* expression in the hypothalamus. In addition, spexin expressed in the ovaries plays a role in oocyte maturation and ovulation by inhibiting GCs proliferation and E2 secretion.

## 6 Discussion and prospects

**Figure 2:**
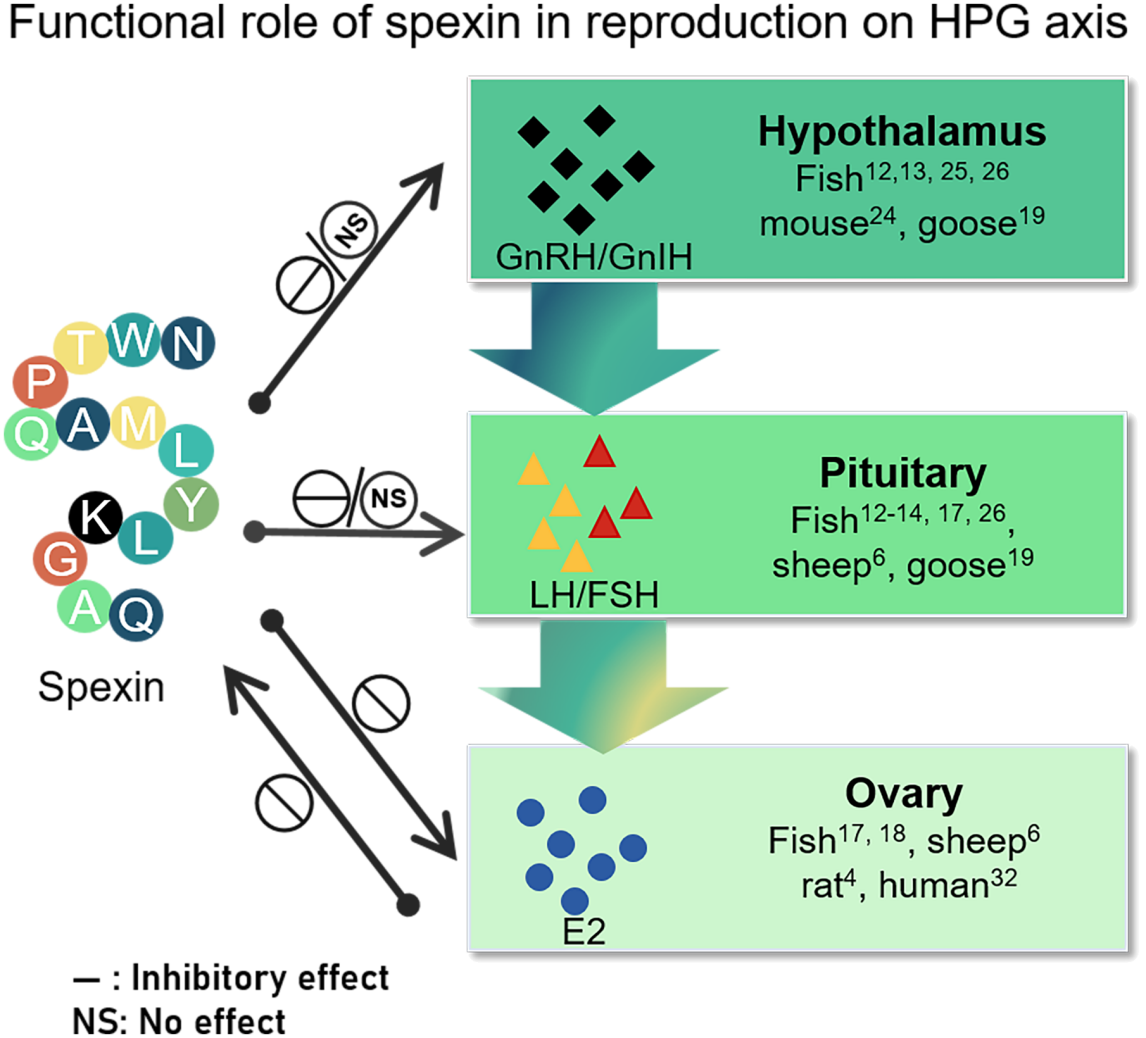
Schematic representation of the role of spexin on the different nodes of HPG and in turn regulated by E2. The effect on HPG axis including its modulation of gonadotropin-releasing hormone, gonadotropin synthesis and secretion, and ovarian function embodying in E2 release. “NS” indicates that spexin has no effect on the different nodes of the HPG axis pointed by the arrow, while “—” indicates that it has an inhibitory effect on the content pointed by the arrow. GnRH, gonadotropin releasing hormone; GnIH, gonadotropin inhibitory hormone; FSH, follicle stimulating hormone, LH, luteinizing hormone; E2, estradiol.

Typically, spexin, which acts as a cognate ligand for GALR2/3, may play an important role in regulating reproductive performance by acting independently or holistically at different nodes of the HPG axis (**Figure 2**). In most teleosts, *spx* expression declines during the breeding season, and gradually diminishes during gonadal development. As an upstream regulator of GnRH neurons, spexin may play a crucial role in modulating GnRH release and physiological activity in the HPG axis. In most species, spexin exhibits either an inactive or inhibitory effect on the HPG, influencing the gene expression of *gnrh* in the brain and decreasing the synthesis and secretion of LH/FSH at both the gene and protein levels. In addition, spexin can act directly on pituitary cells to suppress gonadotropin expression. It can also inhibit ovarian GCs proliferation and E2 release via autocrine or paracrine mechanisms. It is, in turn, regulated by E2.

However, its role in the reproductive capacity remains controversial. Conservatively, the current differential findings of spexin in different animals may be attributed to the species-specific differences, which is probably due to the divergence in reproductive strategies and the presence of redundant reproductive regulatory systems. The disparities might also be attributed to differential results obtained using different methodologies (dose, route of administration, age, and season). Although research on spexin is in its early stages of development, its importance cannot be underestimated. Genes encoding spexin and kisspeptin have been shown to reside in close proximity to the same ancestral chromosome and have co-evolved in species ranging from fish to humans. Kisspeptin regulates GnRH/LH secretion and reproductive behavior. Considering this, the two neuropeptides are hypothesized to exhibit a certain degree of functional overlap in reproduction. Interestingly, kisspeptin is degraded and lost in birds (33, 34), suggesting that spexin may serve as a potential alternative for regulating avian reproduction. To date, the literature on spexin-mediated regulation of reproductive function on different vertebrates is limited. Hence, to obtain a better understanding and in-depth knowledge of spexin, it is necessary to expand these investigations to other species and examine the receptor-activated signal transduction pathways of spexin.

## Author contributions

XC: Writing – original draft. YF: Writing – review & editing. SD: Writing – review & editing. BG: Writing – review & editing. LY: Writing – review & editing. JL: Writing – review & editing. HZ: Writing – review & editing.
